# More than 40 Years Active in Steroid and Isoprenoid Research—A Personal Note on W. David Nes’ Career and His Multiple Achievements in this Field

**DOI:** 10.3390/molecules24050901

**Published:** 2019-03-05

**Authors:** Thomas J. Bach

**Affiliations:** IBMP, CNRS UPR 2357, Université de Strasbourg, Institut de Botanique, F-67000 Strasbourg, France; bach@unistra.fr

W. David Nes has worked on steroids and related fields (lipids!) for more than 40 years, with an enormous scientific output, which is ever increasing and has never been interrupted regardless of where he worked. He is a perfect candidate for the dedication of articles, not only for his “pure science” and related achievements, but also his activity in organizing meetings within societies like the American Chemical Society and especially the American Oil Chemists’ Society (for instance, several “Steroid meetings!”), as co-organizer of plant lipid meetings, and as an active speaker in the “TERPNET” community. He is also author or editor of standard works like *Lipids in Evolution* (with William R. Nes) and *Biochemistry and Function of Sterols* (with E.J. Parish; CRC Press 1997), and a leading member of evaluation panels (e.g., as deputy at the National Science Foundation in Washington, DC for about two years). 

I came into contact with David Nes in 1982, when we detected a common interest in a pathway first described by George Popják (FRS) for mammalian tissue; specifically, the retro-conversion of isopentenyl diphosphate (IPP) into acetyl-CoA via methylcrotonyl-CoA, *trans*-methylglutaconyl-CoA and hydroxymethylglutaryl-CoA, a pathway the amino acid leucine also enters during its degradation. Within essentially six weeks of intense work at the USDA Western Regional Research Center (WRRC) in Albany in early 1983 we could provide clear evidence for such a “mevalonate shunt” pathway in wheat seedlings. A funny remembrance worth mentioning is our sitting side-by-side in darkness at the bench, with green lamps on our heads, to work up etiolated wheat seedlings for incorporation and other experiments, as plants don’t “see” green light.

As a DAAD postdoc fellow I was certainly the first foreign collaborator at a senior postdoc level working with David Nes already in early 1983, being considerably older than he was at that time. It was clear that as a rising star within the USDA Western Regional Research Center in Albany, he had the full support of its director, especially when it came to the use of analytical platforms, from which I greatly profited too. 

From Albany, David Nes then moved to the USDA Richard B. Russell Research Center in Athens, where I could visit him shortly and again discuss scientific matters of interest. A former Humboldt fellow with me (Dr. M. Venkatramesh), at that time still at the Technical University of Karlsruhe (Germany) before my transfer to Strasbourg, joined his lab in Athens and stayed for a while with David Nes when he left the USDA and joined Texas Tech University (TTU) in Lubbock, where he continued his career and successful work until today.

What is always impressive to see from the outside is David Nes’ combination of organic chemistry, the refined analysis of steroids and triterpenoids plus their derivatives, with a deep understanding of biological processes in plants, microorganisms and parasites, including the use of modern methods in molecular biology to elucidate enzymatic reaction mechanisms, and the rational design and synthesis of mechanism-based inhibitors of sterol biosynthesis. These studies led to important clues for sterol evolution across kingdoms, and to chemotherapeutic leads to prevent disease by opportunistic parasites dependent on an intact ergosterol pathway. Furthermore, such studies afforded success in engineering soybean plants with modified sterol seed compositions to benefit human health.

In some more detail, the main focus of David Nes’ research has been to unravel relationships between sterol biosynthesis and function in a range of organisms by unearthing the molecular libraries (genome–metabolome congruence) associated with phyla-specific reaction sequences that regulate the sterol patterning in organisms. Particular emphasis is given on the characterization of intracellular metabolites and enzyme specificities involved in sterol production and processing, and factors regulating the carbon flux and sterol homeostasis. Of course, such studies require collaboration with colleagues and their laboratories (Michael R. Waterman (Nashville), Steven L. Kelly (Swansea), Jonathan Gershenzon (Jena), and Henry T. Nguyen (Columbia). Those collaborations are attested by numerous co-authored publications, the fruits of sabbaticals abroad. 

David Nes is not only known as a very productive scientist and teacher at the university level, but is instrumental in all that concerns “organization and administration”, which is already visible through his functions as Director of the TTU Center for Chemical Biology and Chair of Biochemistry Division, and as Professor of Immunology and Molecular Microbiology at the TTU Health Sciences Center School of Medicine in Lubbock, Texas. This in addition to his activities as associate editor of a number of journals in the field. I would also stress his success in attracting research money for equipment and (international) personnel, not an easy task nowadays.

I wish David Nes all the best for his 65th birthday and for the successful continuation of his research.

